# Antimicrobial resistance and population genomics of emerging multidrug-resistant *Salmonella* 4,[5],12:i:- in Guangdong, China

**DOI:** 10.1128/msystems.01164-23

**Published:** 2024-05-15

**Authors:** Ruan-Yang Sun, Liang-Xing Fang, Jing-Jing Dai, Kai-Chao Chen, Bi-Xia Ke, Jian Sun, Chang-Wen Ke, Edward Wai Chi Chan, Ya-Hong Liu, Sheng Chen, Xiao-Ping Liao

**Affiliations:** 1 National Risk Assessment Laboratory for Antimicrobial Resistance of Animal Original Bacteria, South China Agricultural University, Guangzhou, Guangdong, China; 2 Guangdong Provincial Key Laboratory of Veterinary Pharmaceutics Development and Safety Evaluation, South China Agricultural University, Guangzhou, Guangdong, China; 3 Guangdong Laboratory for Lingnan Modern Agriculture, Guangzhou, Guangdong, China; 4 Department of Food Science and Nutrition, Faculty of Science, The Hong Kong Polytechnic University, Kowloon, Hong Kong, China; 5 Guangdong Provincial Center for Disease Control and Prevention, Guangzhou, Guangdong, China; 6 Jiangsu Co-Innovation Center for the Prevention and Control of Important Animal Infectious Diseases and Zoonoses, Yangzhou University, Yangzhou, Jiangsu, China; Zhejiang University School of Medicine, Hangzhou, Zhejiang, China

**Keywords:** *Salmonella *4,[5],12:i:-, ST34, population genomics, evolution, MDR

## Abstract

*Salmonella* 4,[5],12:i:-, a monophasic variant of *Salmonella* Typhimurium, has emerged as a global cause of multidrug-resistant salmonellosis and has become endemic in many developing and developed countries, especially in China. Here, we have sequenced 352 clinical isolates in Guangdong, China, during 2009–2019 and performed a large-scale collection of *Salmonella* 4,[5],12:i:- with whole genome sequencing (WGS) data across the globe, to better understand the population structure, antimicrobial resistance (AMR) genomic characterization, and transmission routes of *Salmonella* 4,[5],12:i:- across Guangdong. *Salmonella* 4,[5],12:i:- strains showed broad genetic diversity; Guangdong isolates were found to be widely distributed among the global lineages. Of note, we identified the formation of a novel Guangdong clade (Bayesian analysis of population structure lineage 1 [BAPS1]) genetically diversified from the global isolates and likely emerged around 1990s. BAPS1 exhibits unique genomic features, including large pan-genome, decreased ciprofloxacin susceptibility due to mutation in *gyrA* and carriage of plasmid-mediated quinolone resistance (PMQR) genes, and the multidrug-resistant IncHI2 plasmid. Furthermore, high genetic similarity was found between strains collected from Guangdong, Europe, and North America, indicating the association with multiple introductions from overseas. These results suggested that global dissemination and local clonal expansion simultaneously occurred in Guangdong, China, and horizontally acquired resistance to first-line and last-line antimicrobials at local level, underlying emergences of extensive drug and pan-drug resistance. Our findings have increased the knowledge of global and local epidemics of *Salmonella* 4,[5],12:i:- in Guangdong, China, and provided a comprehensive baseline data set essential for future molecular surveillance.

**IMPORTANCE:**

*Salmonella* 4,[5],12:i:- has been regarded as the predominant pandemic serotype causing diarrheal diseases globally, while multidrug resistance (MDR) constitutes great public health concerns. This study provided a detailed and comprehensive genome-scale analysis of this important *Salmonella* serovar in the past decade in Guangdong, China. Our results revealed the complexity of two distinct transmission modes, namely global transmission and local expansion, circulating in Guangdong over a decade. Using phylogeography models, the origin of *Salmonella* 4,[5],12:i:- was predicted from two aspects, year and country, that is, *Salmonella* 4,[5],12:i:- emerged in 1983, and was introduced from the UK, and subsequently differentiated into the local endemic lineage circa 1991. Additionally, based on the pan-genome analysis, it was found that the gene accumulation rate in local endemic BAPS 1 lineage was higher than in other lineages, and the horizontal transmission of MDR IncHI2 plasmid associated with high resistance played a major role, which showed the potential threat to public health.

## INTRODUCTION

Non-typhoidal *Salmonella* (NTS) is an important foodborne pathogen that results in 59,000 deaths and over 4 million disability-adjusted life years lost per annum worldwide (1). Currently, there are over 2,600 serovars of *Salmonella* that have been identified, with a significant number of them posing a potential threat as pathogens. *Salmonella enterica* serovar Typhimurium is one of the most commonly detected serovars in the past decades and also the major causative agent of gastrointestinal infections, especially in immunocompromised individuals, young children, and the elderly (2). A monophasic variant of *Salmonella* Typhimurium, *Salmonella* 4,[5],12:i:-, has been spread worldwide in the last two decades and has become a significant public health concern due to its ability to exhibit multidrug resistance. *Salmonella* 4,[5],12:i:- represents an emerging serotype antigenically related to *S.* Typhimurium (1,4,[5],12:i:1,2), but lacks the second phase flagellar antigen encoded by the *fljB* gene (3). Since the early and mid-1990s, *Salmonella* 4,[5],12:i:- likely diverged from the ancestral Typhimurium and has been increasingly isolated from clinical samples and various animal sources, especially the food-producing animal and food products. Such strain has become detectable in countries ranging from Southeast Asia to North America (4), and has become one of the five most common *Salmonella* serotypes responsible for causing human and animal infections in European countries and the USA (5), suggesting that rapid global emergence and transmission of this multidrug resistance (MDR) serovar across the food chain has occurred, posing an increasing threat to human health.

There were three main distinct clones (i.e., Spanish clone, United States clone, and European clone) that were prevalent in different continents and time periods in history; however, the European clone belonging to sequence type 34 (ST34) has replaced other clones and has become more prevalent around the world, whose population has increased during the last decades (6). The success of this ongoing pandemic has been attributed to resistance to antibiotics and heavy metal. (7). ST34 *Salmonella* 4,[5],12:i:- isolates have been identified to be MDR, implying co-resistance to the four former first-line antibiotics, ampicillin, streptomycin, sulfonamides, and tetracycline (6, 8, 9). ST34 *Salmonella* 4,[5],12:i:- was investigated extensively in high-income settings, including the UK, USA, Japan, and Australia (8, 10, 11). In recent years, ST34 *Salmonella* 4,[5],12:i:- was also the dominant serotype in developing countries in Asia, such as China and Vietnam (12, 13). In China, the proportion of *Salmonella* 4,[5],12:i:- of human origin showed an increased trend since 2011, and exceeded the proportion of Typhimurium in 2016 (14). Alarmingly, multiple MDR transferable plasmids such as IncC and IncHI2 plasmid mediating resistance to first-line and last-line antibiotics such as third-generation cephalosporins, fluoroquinolones (FQs), and colistins were frequently detected in ST34 *Salmonella* 4,[5],12:i:- isolates from several parts of Asia (8, 15, 16). Moreover, despite horizontal transfer of antibiotic resistance genes (ARGs), mutations in the chromosomal quinolone resistance-determining regions (QRDRs) confer FQ resistance and act synergistically with multiple ARGs which have been reported across South East Asia and East Asia. This development significantly limits antimicrobial therapeutic options for severe human salmonellosis in low- and middle-income countries (17–19), posing a threat to future use of drugs for NTS infection treatment.

According to Guangdong Provincial Center for Disease Control and Prevention (CDC), during the past decade, *Salmonella* 4,[5],12:i:- has emerged as the top serovar responsible for causing salmonellosis among diarrhea patients. However, there is limited information regarding the epidemiology of this serotype in Guangdong, China. Herein, we study the epidemic status of *Salmonella* 4,[5],12:i:- isolates over 10 years in Guangdong province and perform WGS analysis of 352 human *Salmonella* 4,[5],12:i:- isolates. In combination with global contextual genomes, we show a thorough scaled appraisal of the population structure, AMR genomic characterization, and transmission routes of *Salmonella* 4,[5],12:i:- across Guangdong, China.

## RESULTS

### Strain collection, AMR phenotype, and ARG distribution

**Fig 1:**
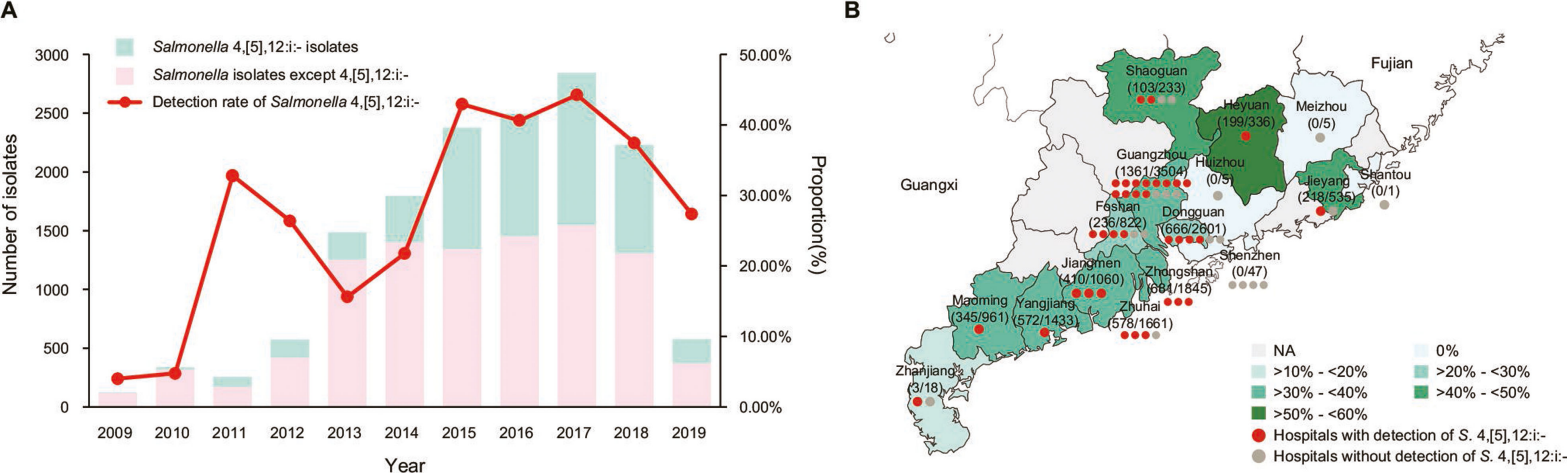
The epidemic of clinical *Salmonella* 4,[5],12:i:- in Guangdong. (**A**) The number of *Salmonella* isolates from diarrheal patients’ infections in Guangdong during 2009 to 2019. The bars depict the total number of *Salmonella* spp. and *Salmonella* 4,[5],12:i:-; the red line depicts the detection rate of *Salmonella* 4,[5],12:i:- isolated by year. (**B**) Geographic distribution and prevalence of *Salmonella* 4,[5],12:i:- isolates across 55 tertiary hospitals in 16 cities within Guangdong Province, China. The map was rendered from coordinates provided in Guangdong provincial standard map service subsystem.

A total of 5,372 *Salmonella* 4,[5],12:i:- isolates were recovered from 15,091 *Salmonella* strains collected from Guangdong Provincial CDC during 2009–2019 in Guangdong Province, China. There was a significant increase in cases of *Salmonella* 4,[5],12:i:- detection from 4.00% in 2009 to 42.95% in 2015. Although it has slightly decreased since 2016, the detection rate still remains above 25% (Fig. 1A). *Salmonella* 4,[5],12:i:- has been identified in 36 tertiary hospitals across 12 cities in Guangdong Province. Among these cities, Heyuan City exhibited the highest detection rate, accounting for 59.23% (Fig. 1B). The male patients accounted for 58.75%, and the median age of these patients was 1 year old (range: neonates to 88 years old). Notably, *Salmonella* 4,[5],12:i:- predominantly affected children under the age of 5, constituting 90.74% of all cases. This observation aligns with the typical patient profile associated with *Salmonella* infection cases. A total of 352 of 5,372 clinical isolates were selected to be sequenced based on sampling cities (approximately 10% of strains randomly selected from each city) and year of isolation to ensure that we had sequencing data available for all study periods (Table S1).

Antimicrobial susceptibility test results showed that 352 clinical *Salmonella* 4,[5],12:i:- strains exhibited high resistance rate to tetracycline (90.1%) and ampicillin (88.6%); moderate resistance to florfenicol (65.3%), chloramphenicol (64.5%), aztreonam (61.6%), sulfamethoxazole/trimethoprim (54.5%), gentamicin (51.7%), and nalidixic acid (50.0%); low resistance to fosfomycin (20.5%), amikacin (7.7%), and tigecycline (0.9%); and sensitivity to meropenem (Fig. S1A; Table S2). In particular, the resistance rate to the first-line antibiotics such as cefotaxime, ceftriaxone, and ceftazidime were 48.9%, 42.6%, and 24.1%, respectively. For ciprofloxacin, 35.2% of the isolates exhibited resistance, with 20 strains exhibiting high resistance level (minimal inhibitory concentrations [MICs] >8 mg/L). Among the tested strains, AMR was relatively common, with 77.3% of the *Salmonella* 4,[5],12:i:- isolates containing MDR profiles and 44.6% carrying extensive drug resistance profiles (Fig. S1B).

The draft genomes of 352 isolates were screened for known genetic determinants of AMR, including horizontally acquired ARGs and mutations within QRDRs. For FQ resistance, 27.6% of isolates were found to have single mutational change in the *gyrA* gene, including D87N (*n* = 80), D87Y (*n* = 13), S83F (*n* = 3), and S83Y (*n* = 1), respectively. Furthermore, seven PMQR genes were detectable in 238 isolates (67.6%), including *oqxAB* (*n* = 123), *qnrS* (*n* = 100), *aac(6’)-Ib-cr* (*n* = 72), *qnrA* (*n* = 1), *qnrB* (*n* = 1), *qnrD* (*n* = 1), and *qnrVC* (*n* = 1). The *gyrA* mutation and PMQR genes were frequently identified in *Salmonella* 4,[5],12:i:- strains, which could be a reason underlying observation of high-proportion ciprofloxacin resistance among such strains. In contrast, third-generation cephalosporin resistance was comparatively less common. Six *bla*_CTX-M_ subtypes were identified, with *bla*_CTX-M-14_ (77/352, 21.9%) and *bla*_CTX-M-55_ (71/352, 20.2%) being more prevalent. The AmpC beta-lactamase-encoding gene *bla*_CMY-2_ (3/352, 0.9%) and other beta-lactamase genes including *bla*_TEM_ (215/352, 61.1%) and *bla*_OXA_ (100/352, 28.4%) have also been detected. In addition, 13 isolates contained *mph*(A) encoding gene conferring azithromycin resistance. Other important ARGs were also observed among these isolates, including *floR* (190/352, 54.0%), *fosA3* (74/352, 21.0%), *mcr-1* (90/352, 25.6%), *mcr-3* (10/352, 2.8%), and *rmtB* (1/352, 0.3%) (Fig. S1C).

### Population structure of ST34 *Salmonella* 4,[5],12:i:- strains

**Fig 2:**
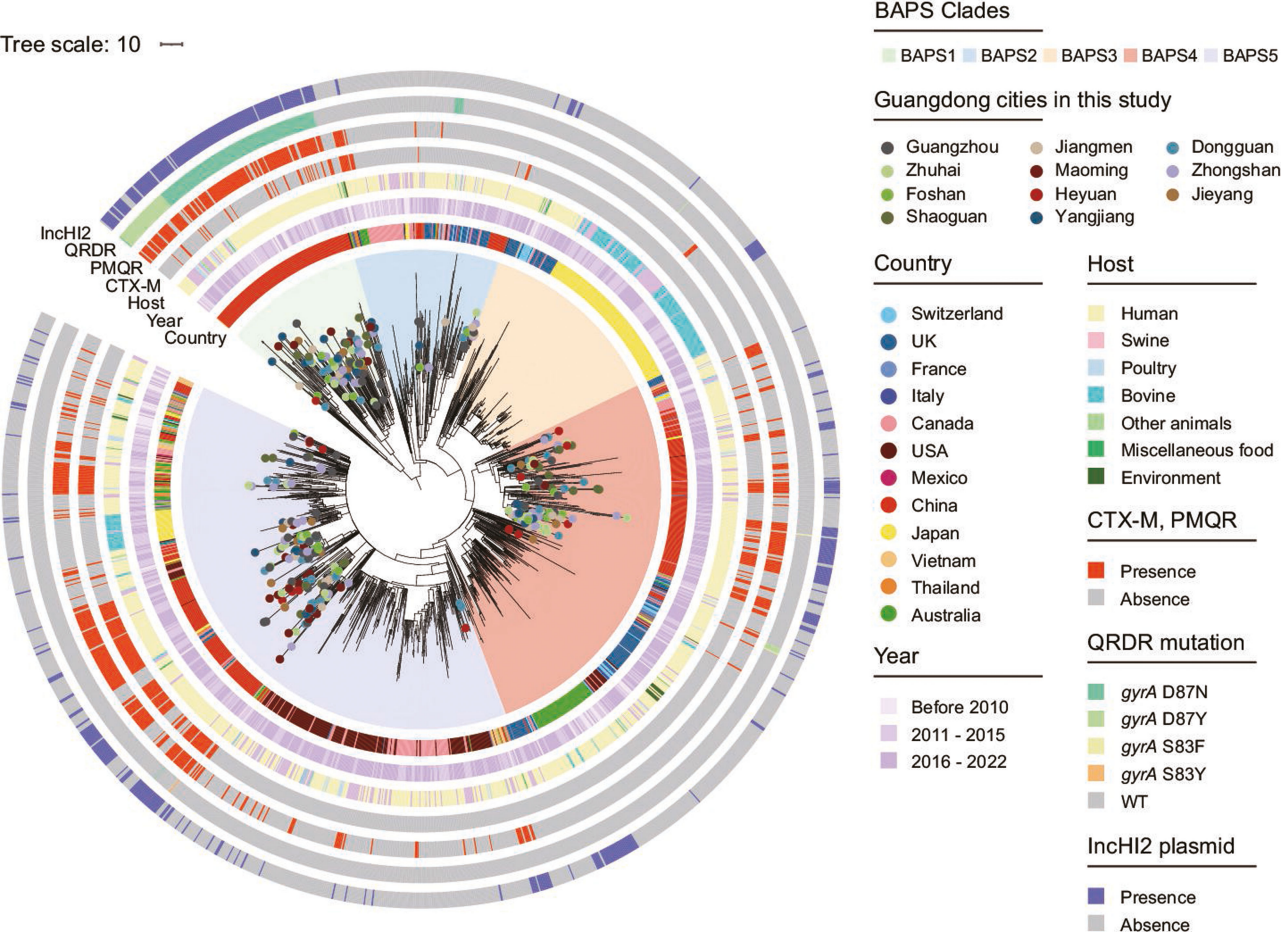
Population structure of ST34 *Salmonella* 4,[5],12:i:-. Maximum-likelihood phylogenetic tree based on the 403 genome sequences reported in this study and 1,142 publicly available ST34 *Salmonella* 4,[5],12:i:- strains with genotypic information and metadata. Assembled reads were mapped to *Salmonella* 4,[5],12:i:- SO4698-09 (National Center for Biotechnology information [NCBI] accession: LN999997.1). The tree is based on 16,584 chromosomal SNPs. The scale bar represents the number of substitutions per site per genome. Tree nodes are colored by country of origin. Metadata is visualized on the concentric rings in compliance to the legend, from inside to outside; 1. year of isolation, 2. source of isolation, 3–5. presence of multidrug resistance markers (CTX-M, PMQR, and QRDR mutation), 6. presence of IncHI2 replicon.

*In silico* multilocus sequence typing (MLST) clustered the 352 clinical *Salmonella* 4,[5],12:i:- strains into five known STs. Among them, ST34 (344/352, 97.73%) was the dominant ST. To further investigate the genetic relationship of ST34 *Salmonella* 4,[5],12:i:- clinical strains in Guangdong, 344 clinical strains from the current study as well as 59 isolates from livestock in our WGS collection, contextualized with 1,142 publicly available isolates from multiple geographical regions, were added into further analysis (Table S3). A recombination-filtered core-genome maximum-likelihood (ML) phylogeny was generated based on an single-nucleotide polymorphism (SNP) alignment of 16,584 bp (Fig. 2). Bayesian analysis of population structure (BAPS) analysis has identified five BAPS groups (Fig. S2). The ST34 *Salmonella* 4,[5],12:i:- genetic structure in Guangdong, China, showed diversity of lineages, with isolates distributed across four lineages except BAPS3 (33.7% in BAPS1, 6.4% in BAPS2, 26.5% in BAPS4, and 42.4% in BAPS5) and occupying basal branches in each lineage.

Interestingly, 116 isolates from 11 Guangdong cities from 2009 to 2019 in this study combined with 36 ones from other provinces in China clustered closely with one isolate from Vietnam formed a new Guangdong lineage (BAPS1 group) and exhibited geographic specificity in the global bacterial population. Furthermore, the partitioning between each BAPS clade was confirmed by core-genome MLST (cgMLST) scheme (Fig. S3), bolstering the identification of a novel Guangdong-associated BAPS clade. The BAPS1 group was found to be different from the other BAPS groups in core-genome SNP (cgSNP)-based comparisons and showed high degree of genetic diversity, with a median pairwise SNP difference of 84. Conversely, other lineage isolates were considerably less diverse, with the median difference within the other lineages being 26–75 SNPs (Table S4; Fig. S4). BAPS1 group strains isolated from various hosts (human, swine, chicken, etc.) persisted within a broad timeframe (2008 to 2021), demonstrating that the Guangdong isolates formed a local lineage distinct from representative isolates of diverse geographical regions and that long-term local reservoirs have been established. The most striking feature of this lineage is the MDR genotype. The strains in BAPS1 contained numerous ARGs (mean, *n* = 16.61), which were frequently conferring resistance to front-line antimicrobials in clinical therapy (Fig. S5). Moreover, the BAPS1 isolates had a much higher occurrence (100%) of single mutational change in the *gyrA* when compared to the other BAPS groups (1.08%). The distribution pattern of *gyrA* substitutions within BAPS1 indicates that these mutations have arisen independently on multiple occasions, confirming that this region of *gyrA* is under strong selection pressure. Meanwhile, the distribution of PMQR genes in BAPS1 was also significantly higher than that of other BAPS groups, yet CTX-M was less common in BAPS1. Additionally, 90.8% (139/153) of strains in BAPS1 harbored the IncHI2 type of MDR plasmid, while only 17.1% (238/1,393) from the other lineages possessed the same type of plasmid.

In addition, BAPS4 and BAPS5 were large and more diverse lineages, consisting of 27.6% (426/1,545) and 39.4% (608/1,545) of *Salmonella* 4,[5],12:i:- isolates analyzed here, respectively. These two lineages encompassed all regions represented in our collection, spanning from 2007 to 2021, suggesting a broad geographical spread of these lineages during the last two decades. In contrast, the BAPS2 lineage exclusively encompassed 157 strains from nine countries, spanning the period between 2002 and 2022, indicating BAPS2 has been less successful at global dispersal than the other lineages. ST34 *Salmonella* 4,[5],12:i:- from Guangdong province in each lineage were very closely related. Isolates from various hospitals in different cities formed tight cluster within BAPS4 and BAPS5, with a median pairwise SNP difference of 37 and 49, respectively (Table S4). Moreover, animal isolates from Guangdong were also observed within tight clusters, suggesting a common source of contamination.

There were numerous instances of very closely related isolates between Guangdong and different European and American countries, which indicate likely transfer events and international dissemination. For example, in BAPS4 lineage, nine isolates sampled from clinical setting isolated in six Guangdong cities between 2014 and 2018 differed by 5–10 SNPs from a human isolate from Canada in 2017. Similarly, in BAPS5 lineage, two isolates sampled from human isolated in Guangzhou in 2009 and 2011 differed by 10 SNPs with an isolate collected in 2010 from food in Thailand. The main difference of these strains in genetic profiles were ARGs that are located in a significant MDR IncHI2 plasmid. These findings suggest that different bacterial populations were epidemic in Guangdong, China; localized expansion of *Salmonella* 4,[5],12:i:- and independent importation of such strains from overseas simultaneously occurred.

### Pan-genome of ST34 *Salmonella* 4,[5],12:i:-

**Fig 3:**
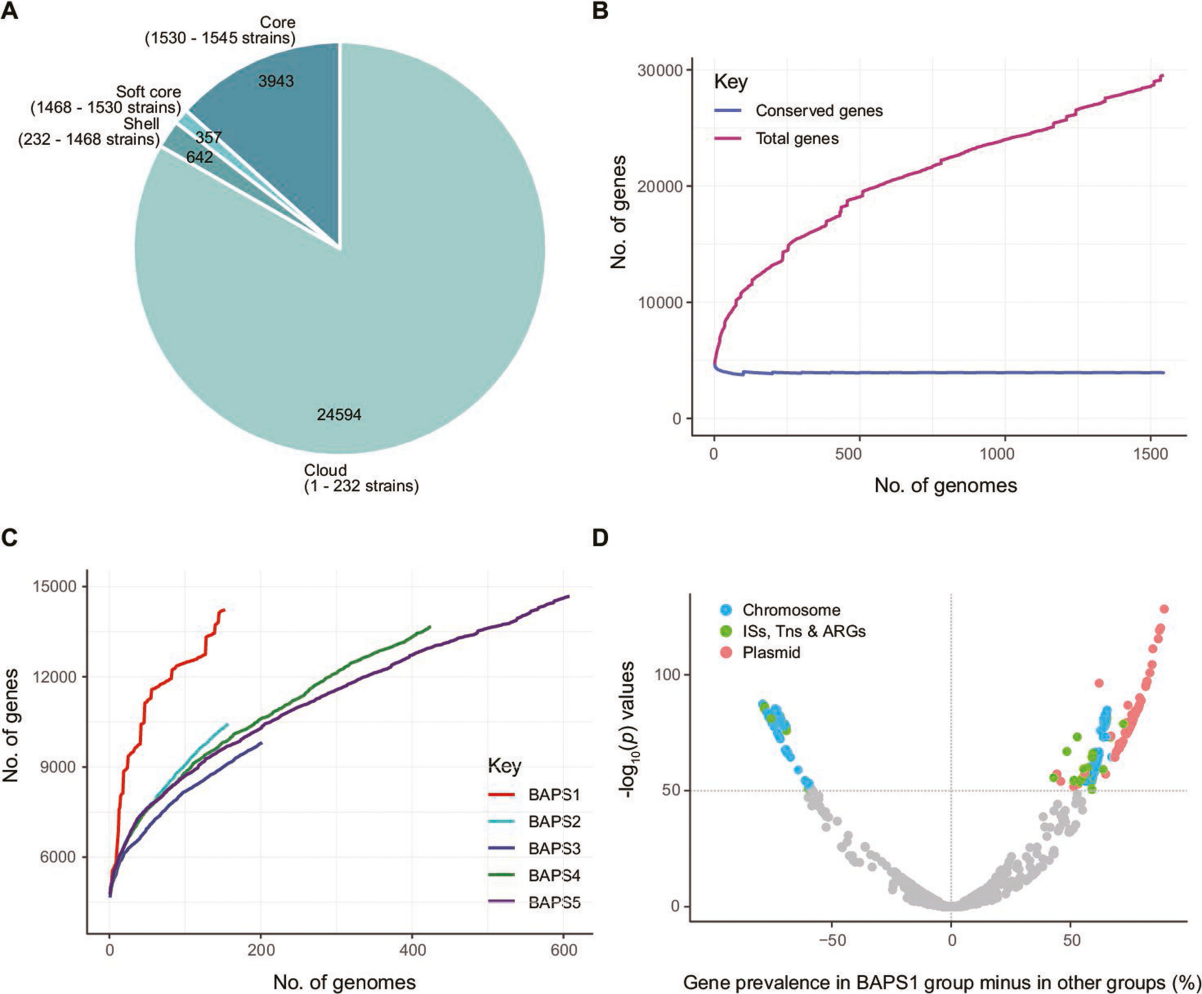
Pan-genome characteristics of ST34 *Salmonella* 4,[5],12:i:- strains. (**A**) Pie charts showing the distribution of core, soft core, shell, and cloud genes. (**B**) Accumulation curves showing the size of the pan-genome, i.e., the totality of unique genes present in *Salmonella* 4,[5],12:i:- (pink line), and the size of the core genome, i.e., genes that are present in at least 99% of the strains (blue line). (**C**) Accumulation curves showing the size of the pan-genome in each BAPS group. (**D**) Volcano plot of different accessory genes between BAPS1 and other BAPS groups. The *y*-axis represents the –log_10_(*P*) values derived from the genome-wide association analysis. The *x*-axis represents the accessory gene prevalence of the population in BAPS1 minus the gene prevalence in other groups.

In order to shed light on the genomic characteristics of *Salmonella* 4,[5],12:i:-, we investigated the core and accessory genome contents within different BAPS clusters. The pan-genome of ST34 *Salmonella* 4,[5],12:i:- consisted of 29,536 orthologous gene clusters, among which the core, soft core, shell, and cloud genes were 3,943, 357, 642, and 24,594, respectively (Fig. 3A). The pan-genome does not appear to be closed, the total number of genes in the pan-genome continued to increase and were not limited by the number of genomes concerned, whereas the core genome remained fairly constant and began to reach a plateau (Fig. 3B).

One key evolution event in local endemic BAPS1 lineage is the acquisition and accumulation of ARGs via the acquisition of IncHI2 plasmids. According to the accumulation curves of the pan-genome, BAPS1 isolates displayed steeper slopes, with an increasing number of sampled genomes that can drastically affect the number of genes in the pan-genomes, whereas isolates from other BAPS groups were found to be more conserved (Fig. 3C). Genome-wide association study (GWAS) analysis indicated that the distribution of accessory gene was specific in BAPS1. Totally, 1,272 accessory genes were carried by 5%–95% of *Salmonella* 4,[5],12:i:- strains, 226 of which were significantly associated with BAPS1 group (Benjamini-Hochberg-adjusted *P* < 1E−50); 136 were located in plasmid, especially the backbone of IncHI2 plasmid, and 30 were mobile genetic elements (MGEs) and ARGs (Table S5). The fact that a substantial proportion of these genes was identified in plasmids suggests that plasmid acquisition contributes to genetic diversification of these isolates in BAPS1 (Fig. 3D).

### Characteristics of mobile elements and the MDR region

**Fig 4:**
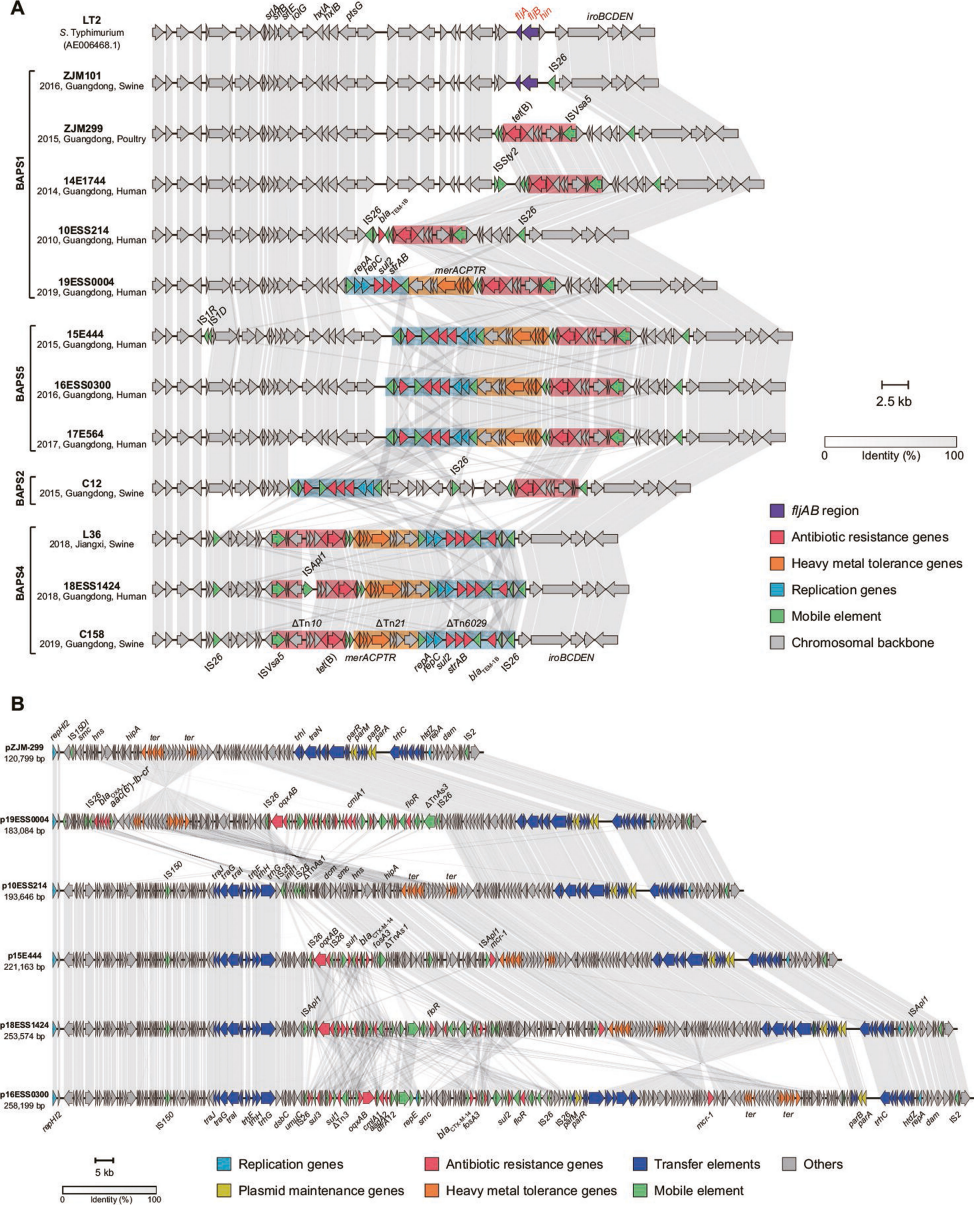
Schematic diagrams of *fljBA* region in the chromosome and IncHI2 plasmid derived from each BAPS lineage, respectively. (**A**) Structural comparison of nature of interruption of the *fljBA* operon. The arrows indicate the direction of gene transcription. Genes are colored according to their sequence and function. A Δ symbol indicates that the gene is truncated. (**B**) Linear comparison of six complete IncHI2 plasmids collected in this study.

In order to explore the accessory gene feature of *Salmonella* 4,[5],12:i:- from each lineage, we selected 12 different strains from different lineages in the phylogenetic tree to obtain the complete genome for better understanding of the co-relationship between MGEs and ARGs in Guangdong isolates. The monophasic phenotypic change of *Salmonella* 4,[5],12:i:- results from the interruption of phase-2 flagellin *fljAB* regions. Chromosomal sequence changes affected the *fljAB* operon and contributed to the monophasic variations. IS*26* played a significant role in mobilization of chromosomal regions by interrupting, deleting, or even replacing the *fljAB* region (Fig. 4A). In ZJM101, an isolate from swine in 2016, IS*26* sequence was inserted between *fljB* and *iroB*, causing the deletion of *hin*, and left the *fljAB* operon intact. This genetic environment represented an early integration event in the ancestral *Salmonella* 4,[5],12:i:- strains. Multiple transposons including Tn*10* (containing tetracycline resistance genes), Tn*21* (containing mercury resistance operon *merACPTR*), and Tn*6029* (containing *bla*_TEM-1_, *sul2*, and *strAB*) were integrated into *fljAB* region via IS*26*. In contrast to BAPS1, BAPS2–5 exhibited more extensive chromosomal interruption by insertion of multiple mobile elements. IS*26*-mediated rearrangement of the *fljAB* operon in BAPS5 is similar to monophasic variants detectable in many European countries (8, 13), indicating that global dissemination of this clone had occurred. In the BAPS2 and BAPS4 lineages, larger inversions (ranging from 25,269 to 32,869 bp) occurred in this region (Fig. 4A). Collectively, these data demonstrate stepwise interruptions of *fljAB*, with a successive accumulation of AMR genes that are distinct across the different BAPS groups of no specific collection year.

In addition to multiple ARGs located on *fljAB* region of the chromosome, it was found that there were diverse types of plasmids carrying ARGs in *Salmonella* 4,[5],12:i:- isolates, including the IncHI2, IncN, IncQ1, and IncF types. In this study, the IncHI2 plasmid was the most frequently observed in *Salmonella* 4,[5],12:i:- isolates in China, accounting for 52.7% of the strains (Tables S1 and S3). IncHI2 plasmids are broad host-range plasmids that contribute to the spread of antibiotic resistance genes and are detectable in specific geographical locations. The prevalence of IncHI2 plasmid has always been significantly higher in China than in the other countries, indicating that independent acquisition of MDR plasmid occurred after the introduction of *Salmonella* 4,[5],12:i:- into China (Fig. S6). Co-occurrence analysis revealed that IncHI2 plasmid plays an important role in the transmission of ARGs in both human and animal hosts, which was significantly and positively correlated with 19 ARGs and 8 insertion sequences (ISs) (*r* > 0.3, *P* < 0.05) (Fig. S7). Among the 12 complete sequenced isolates in this study, several types of IncHI2 plasmids were identified and subjected to further analysis (Table S6).

The sizes of IncHI2 plasmids ranged from 120,799 bp to 258,199 bp with a highly conserved IncHI2-type backbone structure. The core functional genes, including those responsible for plasmid replication, conjugal transfer, maintenance, and stability, were found in all the plasmids. Similar to the chromosome, MDR regions located in IncHI2 plasmids likely evolved through the recombination and integration of a variety of ARGs, such as *bla*_CTX-M-14_, *oqxAB*, *aac(6′)-Ib-cr*, *qnrS1*, *floR*, *fosA3*, and *mcr-1*, with the help of mobile genetic elements, such as ISs and transposons, especially IS*26* (Fig. 4B). Except IncHI2 plasmid, we also obtained four other Inc types of plasmids. The *bla*_CTX-M-55_ was found to be harbored by an IncI1 plasmid, while *mcr-1* was located in the IncX4 plasmid. No other resistance genes were detected in Incp0111 and IncY plasmids.

### Evolutionary and transmission dynamics of ST34 *Salmonella* 4,[5],12:i:-

**Fig 5:**
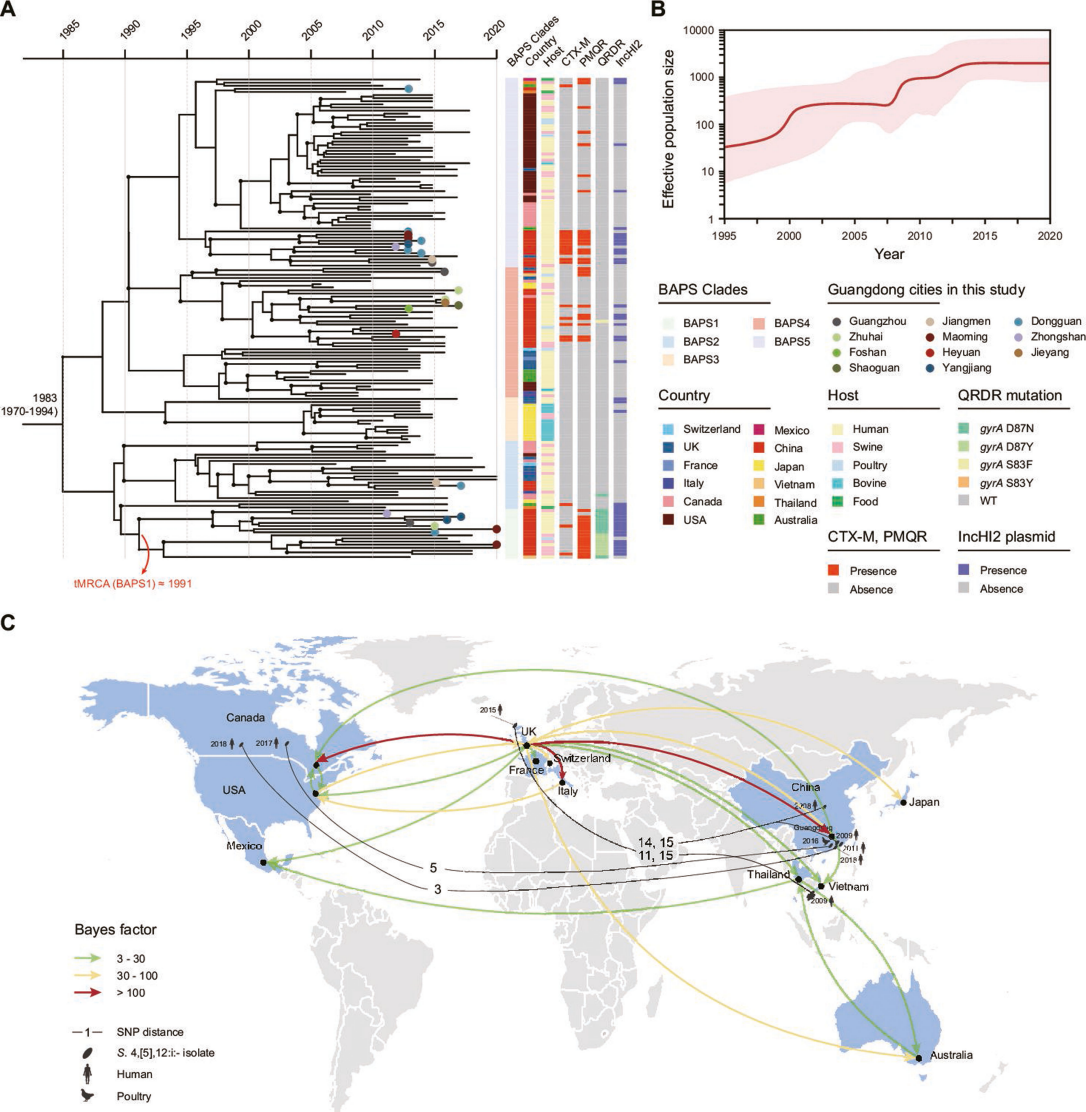
Phylogeographic analysis of ST34 *Salmonella* 4,[5],12:i:- isolates. (**A**) Maximum clade credibility timed tree of global ST34 *Salmonella* 4,[5],12:i:- isolates. The time scale is shown in years. Black dots at branch points indicate posterior probability >90% and dot size is proportional to the posterior probability value. (**B**) Bayesian skyline plot showing the historical changes of the *Ne* of ST34 *Salmonella* 4,[5],12:i:-. The solid red line indicates the median value and the shaded red area represents the 95% HPD of genetic diversity estimates. (**C**) Dissemination routes of ST34 *S*. 4,[5],12:i: across the global data set. The colors of the line indicating statistically significant migration rates are as follows: red line, very strongly significant migration rates with 100 ≤ Bayes factor (BF); yellow line, strongly significant rates with 10 ≤ BF < 100; blue line, significant rates with 3 ≤ BF < 10. The numbers on the solid lines indicate pairwise core-genome SNP distance between the isolate recovered from other countries and each Chinese isolate. The world map was rendered from coordinates provided in rworldmap package in R.

A representative selection of 155 ST34 *Salmonella* 4,[5],12:i:- isolates was subjected to a phylogeographical analysis, aiming to gather further information on the evolutionary history and geographic spread of *Salmonella* 4,[5],12:i:- across Guangdong. Linear regression of root-to-tip distances against year of isolation indicated strong temporal signal for molecular clock analysis (Fig. S8), as did date-randomization tests (Fig. S9). Our analysis estimated the mutation rate was 3.91 × 10^−7^ substitutions per site per year (95% highest posterior density [HPD] = 2.68 × 10^−7^ to 5.25 × 10^−7^), which was equivalent to accumulation of 2.04 SNPs per genome per year (95% HPD = 1.39 to 2.73). This estimated substitution rate was similar to previous estimates for other non-typhoidal *Salmonella* serotypes (Typhimurium and Kentucky) (20, 21). The most recent common ancestor was estimated to have emerged circa 1983 (95% HPD: 1970 to 1994). The topologies of the BEAST and maximum-likelihood trees were congruent, while the local lineage BAPS1 may have emerged around 1991, suggesting a recently derived clone of *Salmonella* 4,[5],12:i:- circulating in Guangdong (Fig. 5A). By reconstruction of the population dynamics over time using the Bayesian skyline model, the effective population size (*Ne*) of ST34 *Salmonella* 4,[5],12:i:- was predicted to undergo three exponential increases from 2000 to 2015, and then remained steady in recent years, coinciding with the *Salmonella* 4,[5],12:i:- pandemic (Fig. 5B) (22).

We then conducted a phylogeographic analysis of the isolates to clarify the transmission features over time. This analysis revealed 21 statistically supported (Bayes factor [BF] >3) routes of transmission among 12 countries (Table S7). UK was likely the epicenter for the spread of ST34 *Salmonella* 4,[5],12:i:-. This country was found to be linked with all the other 11 countries, including Italy (BF = 156), China (BF = 121), Canada (BF = 101), and others, suggesting that ST34 *Salmonella* 4,[5],12:i:- likely emerged in the UK. Thereafter, the strains migrated to other continents and resulted in a stable establishment in China. Other countries linked by possible intercontinental transmission of ST34 *Salmonella* 4,[5],12:i:- include the UK (BF = 35), Canada (BF = 10), and Vietnam (BF = 8.5) (Fig. 5C). In these cases, isolates from the linked countries were phylogenetically close (3–15 SNPs) to *Salmonella* 4,[5],12:i:- collected from this study in Guangdong. However, the lack of a consistent pattern of ARGs and *gyrA* mutation among most closely linked isolates in different countries suggests the acquisition of MDR genes occurred independently in China at a local level.

## DISCUSSION

*Salmonella* 4,[5],12:i:- is one of the most common foodborne pathogens that causes large-scale, national, and international outbreaks and food recalls. The number of *Salmonella* 4,[5],12:i:- cases has notably increased during the past two decades. Until recently, MDR lineages such as ST34 European clone have dominated in Asia, in particular, in China (10, 12, 14). Understanding the population structure and genomic epidemiology of *Salmonella* 4,[5],12:i:- is essential for effective control and prevention of infections caused by this pathogen. Phylogeographic analysis provided new insight into the global population structure of ST34 *Salmonella* 4,[5],12:i:-. We have identified five significant clades and underlined the emergence of both local transmission and importation co-existing mode in the current pandemic. BAPS1 cluster, a large, divergent lineage of strains with a broad host range, circulated predominantly in Guangdong and other provinces in China and had disseminated to Vietnam within the past 30 years. BAPS1 strains, which are distinct from strains in other continents, emerged around 1990s, indicating prolonged local transmission of this clade, suggesting that Guangdong might be another hotspot for emerging subpopulations. This cluster exhibited less diversity in the *fljAB* region, near ubiquitous carriage of MDR IncHI2 plasmids, reduced sensitivity to FQs due to the mutation of *gyrA* and the carriage of PMQR genes, a larger pan-genome, and distinctive accessory gene content. Meanwhile, Guangdong isolates were also broadly disturbed in multiple global lineages. There were numerous instances of very closely related isolates from different countries (number of pairwise cgSNPs less than 15), raising the possibility of intercontinental transmission of the global lineage through international travel and global food product trade. Taken together, these results suggest that highly resistant *Salmonella* 4,[5],12:i:- circulating in Guangdong, China, is positioned within the diverse and polyphyletic *Salmonella* 4,[5],12:i:- population, two independent transmission modes, local dissemination, and intercontinental transmission and circulation co-exist in this country.

Our analysis yields some observations relevant to the origin of *Salmonella* 4,[5],12:i:- from two perspectives: year and country. By Bayesian analysis, Europe, most probably the UK, may be the birthplace and longest established home of *Salmonella* 4,[5],12:i:-. Given the considerable spread and time depth of European isolates, our phylodynamic analyses are most consistent with introduction of *Salmonella* 4,[5],12:i:- into China from the UK, then to Vietnam and Canada. Inter-region transfers based on the maximum-likelihood phylogeny and locations provide clear evidence of several candidate intercontinental transfers. A 2020 study investigated the emergence and global spread of the dominant ST34 lineage in *Salmonella* 4,[5],12:i:-, and subsequent analysis examined the spread of *Salmonella* 4,[5],12:i:- in North America and Europe (10). Our analysis reveals a similar substitution rate with their results, but estimated the emergence of *Salmonella* 4,[5],12:i:- in 1983, 11 years earlier than their observation. This discrepancy may be due to the different sample subsets used in the analysis. The *Ne* is estimated from the observed nucleotide variation (genetic diversity) in relation to the mutation rate and can be used to infer changes in the size of a population (23). The *Ne* of ST34 *Salmonella* 4,[5],12:i:- suggested a nearly 10-fold increase from 2000 to 2010 and remained at a plateau level over the next 10 years. This may partly explain why the ST34 clone subsequently increased since 2000 and still represent >80% of strains documented in the database.

The success of ST34 *Salmonella* 4,[5],12:i:- has been attributed to its MDR feature, and the MDR phenotype of *Salmonella* 4,[5],12:i:- strains is likely influenced by different regional antibiotic usage . Antimicrobial abuse in agricultural and clinical settings favored the development of resistance to different antibiotics in *Salmonella* and then led to the spread of antimicrobial-resistant bacteria/genes (24, 25). Of particular concern is the high prevalence of resistance to those first-line agents currently recommended for the treatment of salmonellosis. In our strain collection, almost 50% of isolates displayed resistance to fluoroquinolone and cephalosporins. Notably, point mutation resistance to fluoroquinolone was significantly more common in Chinese isolates, especially in BAPS1 isolates. Despite chromosome, the genetic basis for MDR was a conjugative (i.e., self-transmissible) plasmid of incompatibility type IncHI2 plasmid. IncHI2 plasmids are plastic and can capture a variety of ARGs through MGEs, which have been considered the main vehicle for resistance to first-line antimicrobials, promoting dissemination of this MDR lineage in China (17, 26–28). The IncHI2 plasmid carriage rate in China was far more higher than any other countries; IncHI2 plasmids were favorably maintained in the *Salmonella* 4,[5],12:i:- following local introductions, despite geographical discrepancy. We found that recent patterns of antibiotic resistance and IncHI2 plasmid carriage among strains in Guangdong have been largely driven by the independent local acquisition of resistance, rather than by resistance determinants already present within the genomes of the imported lineages; Guangdong has been identified as a hotspot where IncHI2 plasmid-driven resistance coincides with chromosomal point mutation (29).

We acknowledge several limitations in this study. Firstly, it was a regional analysis, mainly focused on Guangdong province in China, and the results may not be universal and representative. Secondly, the time span was large while the sample size was relatively small in several years, which leads to little information regarding the timeline changes. Lastly, the numbers of isolates from animals, foods, and the environment were relatively limited, which may result in some missing information, so more surveillance data are needed to accurately reflect the antibiotic resistance of isolates from these sources. Although we included all *Salmonella* 4,[5],12:i:- isolates from publicly available databases, we may still not be able to achieve a sufficiently high confidence level to allow speculation on the geographical spread and local evolution of *Salmonella* 4,[5],12:i:- in Guangdong, China; this limitation could have affected the conclusion.

In conclusion, this study highlights the emergence and geographical spread of *Salmonella* 4,[5],12:i:-, an important MDR public health pathogen. The local expansion and international circulation of *Salmonella* 4,[5],12:i:- co-exist in Guangdong, China, and their ability to acquire AMR genes driven by IncHI2 plasmid, which conferred high-level resistance to first-line and last-line antibiotics. All these factors have jointly promoted the widespread dissemination of this serotype in Guangdong, China, which highlights the need for improved surveillance of novel bacterial pathogens in a One Health context. Continuous monitoring of MDR *Salmonella* 4,[5],12:i:- in animals and humans is required for promoting food safety and improving public health.

## MATERIALS AND METHODS

### Strain collection and antimicrobial susceptibility testing

From 2009 to 2019, Guangdong Provincial CDC obtained 15,091 *Salmonella* isolates from 55 tertiary hospitals located in 16 cities across Guangdong, China, as part of a clinic-based *Salmonella* infection surveillance program for outpatients with diarrhea. A total of 5,372 isolates were identified as *Salmonella* 4,[5],12:i:- according to the Kauffmann-White scheme. Three hundred fifty-two isolates were selected for whole-genome sequencing.

Additionally, our lab compiled a data set of 1,240 *Salmonella* isolates from 2015 to 2021, derived from various animal hosts across 25 provinces and municipalities (Fig. S10). Among these isolates, 232 were identified as *Salmonella* 4,[5],12:i:-. Fifty-nine isolates were selected for whole-genome sequencing.

To provide a global context, additional *Salmonella* 4,[5],12:i:- genomes were downloaded from the NCBI Pathogen database and previous studies (8, 13, 17). Basic information of all genomes was collected from the NCBI by using an automated python script and excluded strains in which any of the metadata such as host, year, or country was missing. At last, *Salmonella* genome was re-confirmed as *Salmonella* 4,[5],12:i:- using *in silico Salmonella* serotyping software SISTR (30). Metadata of whole-genome sequencing data of *Salmonella* 4,[5],12:i:- isolates was collected in Microsoft Excel, listed in Tables S1 and S3. Additionally, the data were visualized in Fig. S11.

The MICs of 18 antimicrobial agents for the *Salmonella* isolates were determined using the agar dilution method and the results were interpreted according to the European Committee on Antimicrobial Susceptibility Testing (v.10.0) and the Clinical and Laboratory Standards Institute document (M100-S28 and VET01-S2) (31, 32). *Escherichia coli* ATCC 25922 served as a quality control strain.

### Whole-genome sequencing and screening

The genomic DNA of tested isolates was extracted using the TIANamp Bacteria DNA Kit (Tiangen, Beijing, China) and subjected to 250 bp paired-end WGS using the Illumina HiSeq System (San Diego, CA, USA). Trimmed reads were assembled by SPAdes v.3.6.2 (33). MLST and cgMLST were analyzed using the MLST tool (https://github.com/tseemann/mlst/) and SeqSphere+ v.3.4.0 (http://www.ridom.de/seqsphere/), respectively. Antimicrobial resistance genes, target mutations in QRDRs, heavy metal tolerance genes, and plasmid replicon types were obtained using AMRFinder v.3.8.4 (34) and ABRicate v.1.0.1 (https://github.com/tseemann/abricate). The network graph describing co-occurrence of ARGs, plasmid replicons, and insertion sequences was constructed using Gephi v.0.9.2 (35).

### Phylogenetic analysis

Since the UK, USA, Canada, and Japan collections are substantially larger than the collection from other countries, for the purpose of reducing computation time, 200 samples from each aforementioned country were randomly selected using a random number generator in Microsoft Excel for corresponding analysis. Assemblies were mapped to the reference sequence *Salmonella* 4,[5],12:i:- SO4698-09 (NCBI accession: PRJEB10340) using Snippy v.4.6.0 (https://github.com/tseemann/snippy). SNPs were called, recombinant regions were removed using Gubbins v.2.4.1, and the final SNPs were extracted with SNP-sites (36, 37). A ML phylogenetic tree was inferred using RAxML v.8.2.12 (GTRGAMMA substitution model) (38). The final tree was mid-point rooted and visualized with iTOL v.5 (39). Pairwise SNPs between strains were counted from the core SNP alignment with snp-dists v.0.7.0. The lineages of the phylogenetic tree were defined using rhierBAPs (40).

### Pan-genome analysis

Prokka v.1.14.6 was used to annotate the assembled genomes (41). Pan-genome analysis was carried out using Roary v.3.13 to cluster the genes encoding complete protein sequences into core (core and soft core) and accessory (shell and cloud) genes (42). Pan-genome-wide association analyses were performed using Scoary v.1.6.16 (43). We defined the BAPs group as traits and used Scoary with the –no_pairwise flag and a Benjamini-Hochberg corrected *P*-value threshold of 1E−50 to identify genes that were over- or under-represented in those clusters. Clusters of orthologous genes were annotated using eggNOG v.5.0 (44).

### Phylogeographic analysis

To ensure that there was no sampling bias, 155 representative isolates distributed across the ML tree and covering the full temporal and geographic range were selected. The temporal signal was investigated in Tempest v.1.5.3 (45). We used BEAST v.1.10.4 (46) to date the important nodes and spatiotemporal dynamics. The concatenated 2,926 non-recombinant chromosomal SNP alignments were subjected to BEAST analyses with the relaxed, uncorrelated lognormal clock model, in combination with Bayesian skyride coalescent model. Markov chain Monte Carlo (MCMC) analyses were run three times with chain lengths of 100 million, sampling every 10,000 generations, and the first 10% of the samples were discarded as burn-in. The estimation of the relevant evolutionary parameters was checked using Tracer v.1.7.1, and a summary tree was produced by TreeAnnotator v.2.6.0. Afterward, the summary tree was processed with SPREAD v.0.9.7 to generate a spatio-temporal diffusion analysis using the sample location data (47). To test the robustness of the molecular clock signal, 20 further BEAST runs with randomized tip dates were generated using the same model (48).

### Long-read sequencing of representative *Salmonella* 4,[5],12:i:- strain

Twelve representative isolates were selected for long-read sequencing using the Oxford Nanopore Technologies (ONT) GridION Platform (Nanopore, Oxford, UK). Isolates were selected for sequencing based on the diversity of AMR profiles and lineage membership. ONT sequencing was performed using the R9.4 flow cell (FLO-MIN106). Raw reads generated by MinKNOW were base-called and barcoded using Guppy. The *de novo* hybrid assembly of both short (Illumina) and long reads (ONT) was performed using Unicycler v.0.5.0 (49). Sequence comparison of chromosome and plasmids was conducted using Clinker v.0.0.25 (50).

## Data Availability

All whole-genome sequencing data have been deposited in the NCBI database BioProject: PRJNA885269 and PRJNA629650. Source data and codes used for AMR analysis, phylogeography analysis and visualization are deposited in Github (https://github.com/RuanyangSun/Salmonella_4-5-12-i--_ST34_China).

## SUPPLEMENTAL MATERIAL

Supplemental figures msystems.01164-23-s0001.docx Fig. S1 to S11.

Table S1 msystems.01164-23-s0002.csv Metadata of the 352 clinical and 59 animal *Salmonella* 4,[5],12:i:- isolates analyzed in this study.

Table S2 msystems.01164-23-s0003.csv Antimicrobial susceptibility testing of 352 *Salmonella* 4,[5],12:i:- strains.

Table S3 msystems.01164-23-s0004.csv Information regarding of 6,433 *Salmonella* 4,[5],12:i:- strains obtained from the NCBI database and previous studies.

Table S4 msystems.01164-23-s0005.csv Pairwise comparison of single nucleotide polymorphisms of 1,546 ST34 *Salmonella* 4,[5],12:i:- isolates.

Table S5 msystems.01164-23-s0006.csv Summary of the genome-wide significant and suggestive accessory genes associated with BAPS1 isolates identified by the GWAS analysis.

Table S6 msystems.01164-23-s0007.csv Details of twelve isolates with complete genome sequences finished by Illumina and ONT sequencing.

Tables S7 msystems.01164-23-s0008.csv Bayes factor test for non-zero rates for global ST34 *Salmonella* 4,[5],12:i:- strains recovered in different geographical regions.

ASM does not own the copyrights to Supplemental Material that may be linked to, or accessed through, an article. The authors have granted ASM a non-exclusive, world-wide license to publish the Supplemental Material files. Please contact the corresponding author directly for reuse.
