# Supplementary material for: Antimicrobial resistance and population genomics of emerging multidrug-resistant Salmonella 4,[5],12:i:- in Guangdong, China
Source: mSystems. 2024 May 15;9(6):e01164-23. doi: 10.1128/msystems.01164-23 (PMC11237462; doi:10.1128/msystems.01164-23)
Supplement: Supplemental figures — Fig. S1 to S11. [file msystems.01164-23-s0001.docx]

Supplementary Materials for

**Antimicrobial resistance and population genomics of emerging multidrug-resistant *Salmonella* 4,[5],12:i:- in Guangdong, China**

Ruan-Yang Sun^1,2#^, Liang-Xing Fang^1,2,3#^, Jing-Jing Dai^1,2^, Kai-Chao Chen^4^, Bi-Xia Ke^5^, Jian Sun^1,2,3^, Chang-Wen Ke^5^, Edward Wai chi Chan^4^, Ya-Hong Liu^1,2,3,6^, Sheng Chen^4*^, Xiao-Ping Liao^1,2,3*^

^1^National Risk Assessment Laboratory for Antimicrobial Resistance of Animal Original Bacteria, South China Agricultural University, Guangzhou, Guangdong, P. R. China.

^2^Guangdong Provincial Key Laboratory of Veterinary Pharmaceutics Development and Safety Evaluation, South China Agricultural University, Guangzhou, Guangdong, P. R. China.

^3^Guangdong Laboratory for Lingnan Modern Agriculture, Guangzhou, Guangdong, P. R. China.

^4^Department of Food Science and Nutrition, Faculty of Science, The Hong Kong Polytechnic University, Kowloon, Hong Kong, P. R. China.

^5^Guangdong Provincial Center for Disease Control and Prevention, Guangzhou, Guangdong, P. R. China.

^6^Jiangsu Co-Innovation Center for the Prevention and Control of Important Animal Infectious Diseases and Zoonoses, Yangzhou University, Yangzhou, Jiangsu, P. R. China.

^#^ These authors contributed equally.

^*^ Corresponding Author: Sheng Chen, Email: sheng.chen@polyu.edu.hk; Xiao-Ping Liao, E-mail: [xpliao@scau.edu.cn](mailto:xpliao@scau.edu.cn).

**Supplemental Tables**

**Table S1. Metadata of the 352 clinical and 59 animal *Salmonella* 4,[5],12:i:- isolates analyzed in this study**

**Table S2.** **Antimicrobial susceptibility testing of 352 *Salmonella* 4,[5],12:i:- strains (MICs, mg/L)**

**Table S3.** **Information regarding of 6,433 *Salmonella* 4,[5],12:i:- strains obtained from the NCBI database** **and previous studies**

**Table S4. Pairwise comparison of single nucleotide polymorphisms of 1,546 ST34 *Salmonella* 4,[5],12:i:- isolates**

**Table S5. Summary of the genome-wide significant and suggestive accessory genes associated with BAPS1 isolates identified by the GWAS analysis**

**Table S6.** **Details of twelve isolates with complete genome sequences finished by Illumina and ONT sequencing**

**Table S7. Bayes factor test for non-zero rates for global ST34 *Salmonella* 4,[5],12:i:- strains recovered in different geographical regions**

**Supplemental Figures**


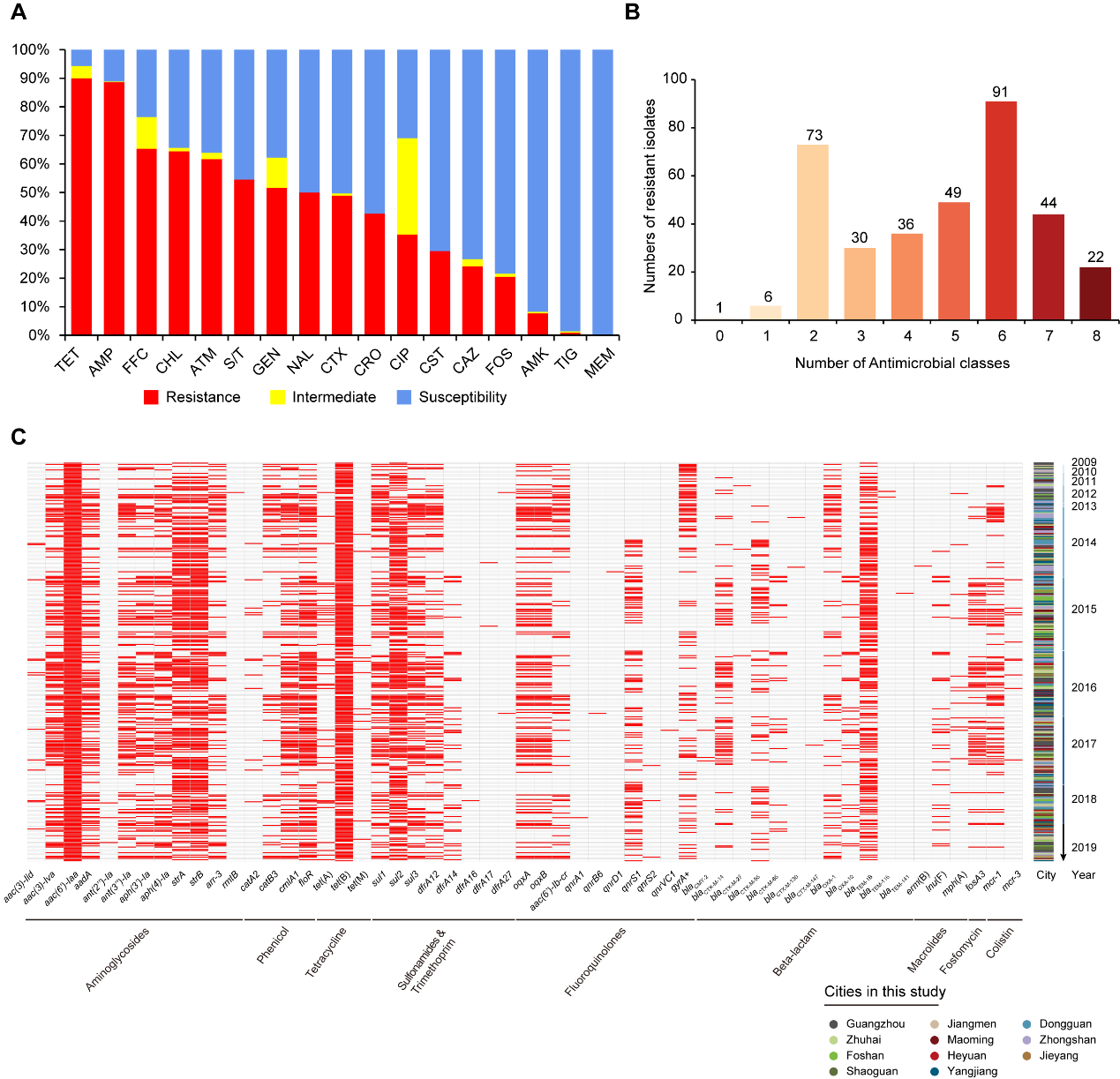


**Figure S1. Antimicrobial resistance characteristics of 352 clinical *Salmonella* 4,[5],12:i:- strains isolated from this study**

**(A)** The percentage of *S*. 4,[5],12:i:- isolates resistant to each of the antibiotics tested;

TET: tetracycline, AMP: ampicillin, FFC: florfenicol, CHL: chloramphenicol, S/T: sulfamethoxazole/trimethoprim, ATM: aztreonam, NAL: nalidixic acid, GEN: gentamicin, CTX: cefotaxime, CRO: ceftriaxone, CST: colistin, CIP: ciprofloxacin, CAZ: ceftazidime, FOS: fosfomycin, AMK: amikacin, TIG: tigecycline, MEM: meropenem.

**(B)** Numbers of *S*. 4,[5],12:i:- isolates resistant to different antibiotic classes tested;

**(C)** Heatmap of antimicrobial resistance genes harbored by 352 clinical *S*. 4,[5],12:i:- in Guangdong, China

Horizontal axes represent the antimicrobial resistance genes and vertical axes represent year of isolation, and city of isolation. The classes of antibiotics to which the genes confer resistance are labelled at the bottom of the figure. Red boxes represent the presence of the corresponding items among sequenced isolates, and white boxes represent their absence.

**
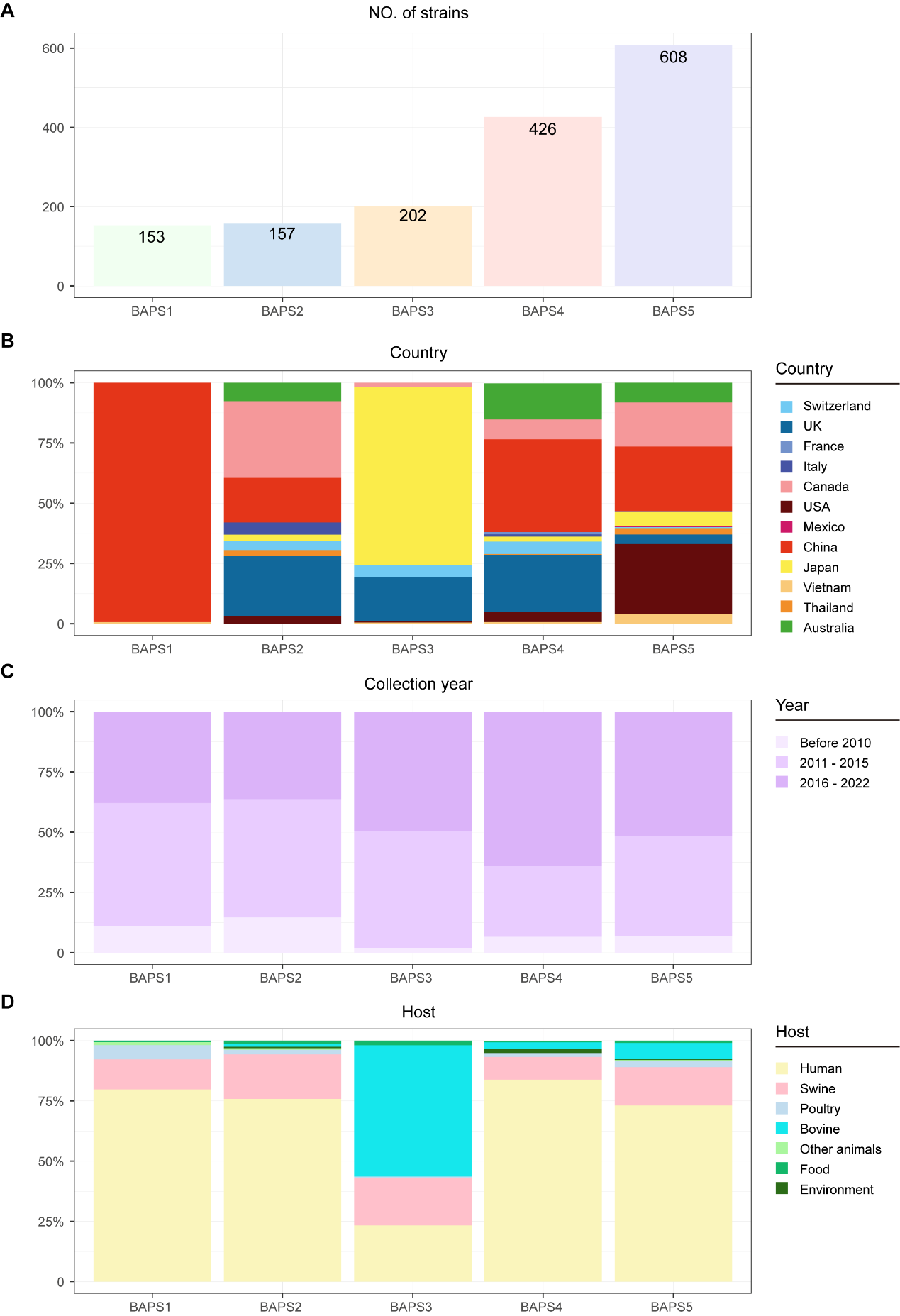
Figure S2.** **Characteristics of ST34 *S*. 4,[5],12:i:- isolates in BAPS cluster**

**(A)** Number of isolates that were clustered in each of the BAPS cluster;

**(B)** Fraction of countries of origin in each BAPS cluster;

**(C)** Fraction of collection year in each BAPS cluster;

**(D)** Fraction of isolates in BAPS clusters isolated from different hosts.

**
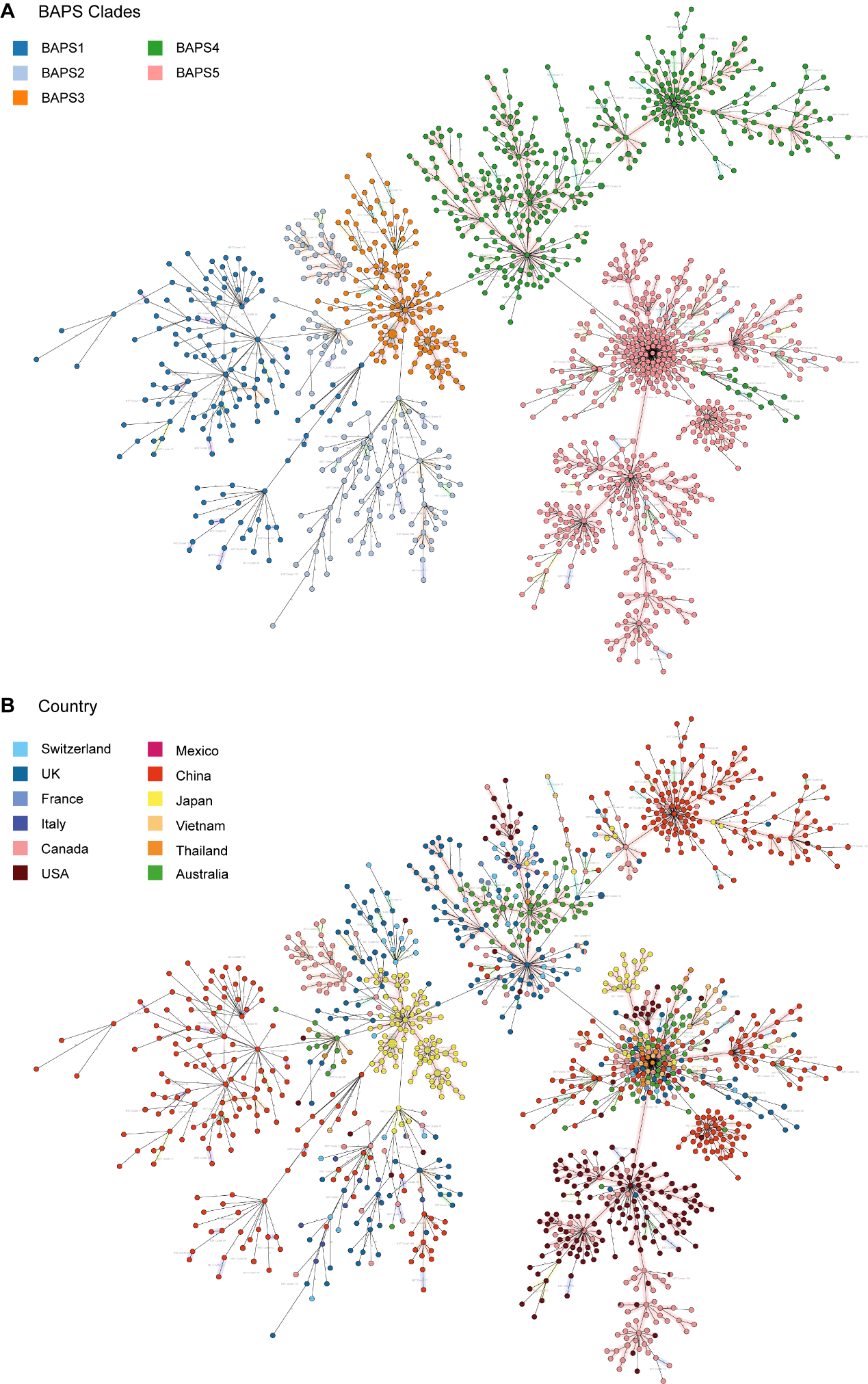
Figure S3. Minimum spanning tree constructed on the basis of cgMLST allelic genes of 1545 ST34 *S*. 4,[5],12:i:-** **isolates** **based on** **BAPS clade (A) and country of origin (B)**

Each circle depicts an allelic profile based on sequence analysis of 3002 cgMLST genes. The size of each node indicates the number of isolates within that node. Isolates are color-coded by BAPS clade and country of origin. The length of the connecting lines represents the number of target genes with different alleles.


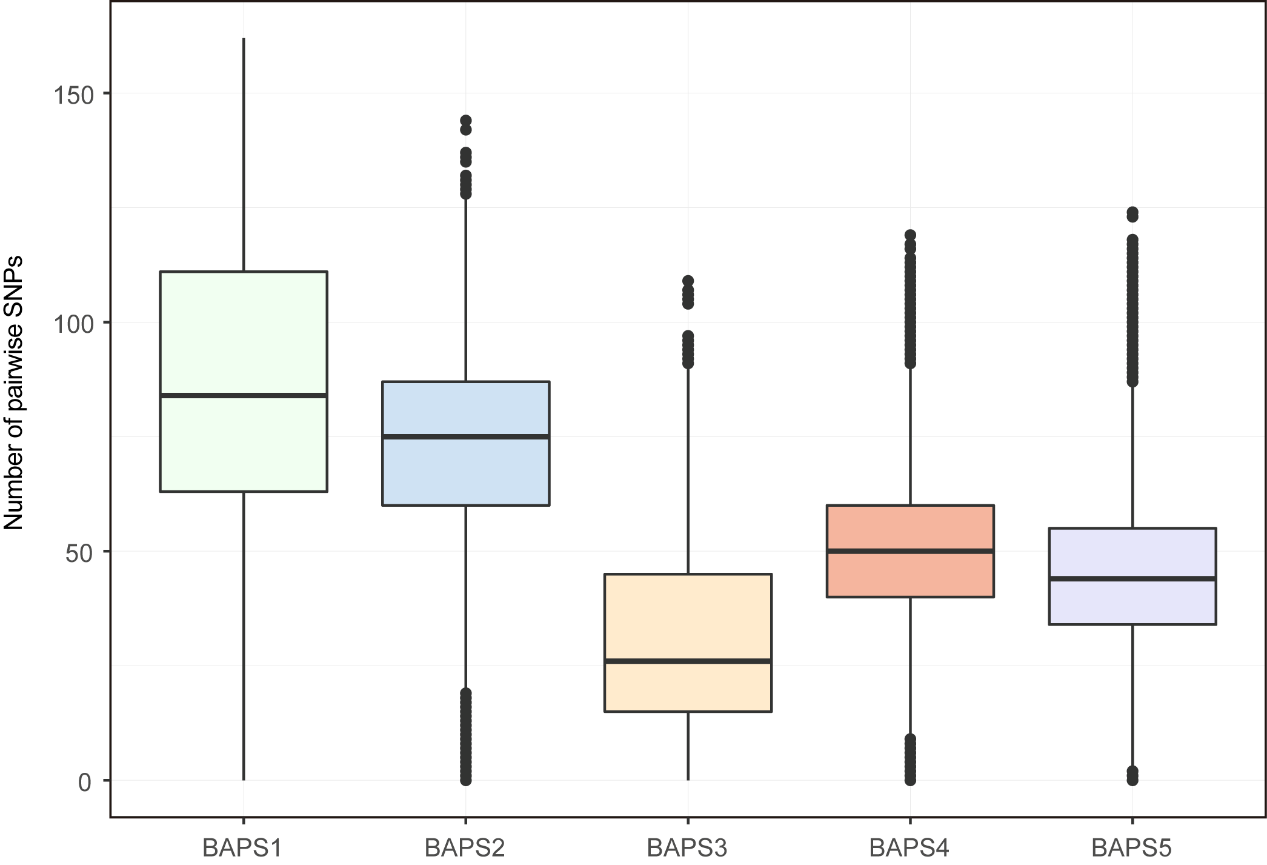


**Figure S4.** **Core-genome pairwise SNP comparisons within each BAPS cluster**

Distribution of pairwise SNPs within each BAPS cluster of *Salmonella* isolates.

**
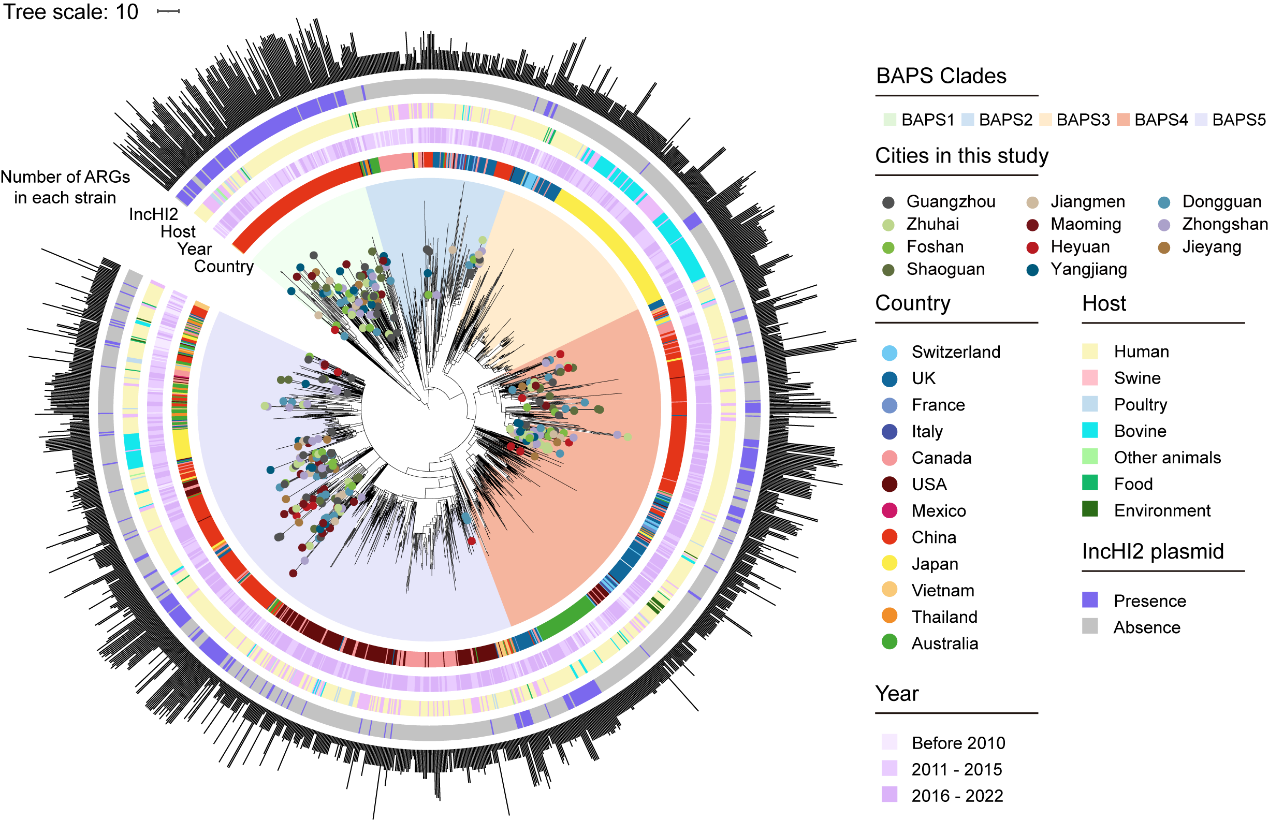
**

**Figure S5.** **Maximum likelihood phylogenetic tree based on the 403 genome sequences of this study and those of 1142 publicly available ST34 *S*. 4,[5],12:i:- strains with genotypic information and metadata**

Tree nodes are colored by country of origin. Metadata is visualized on the concentric rings in compliance to the legend, from inside to outside; 1. Country of isolation, 2. Year of isolation, 3. Source of isolation, 4. Carriage of IncHI2 plasmid, 5. Number of the total ARGs carried by the corresponding bacteria.

**
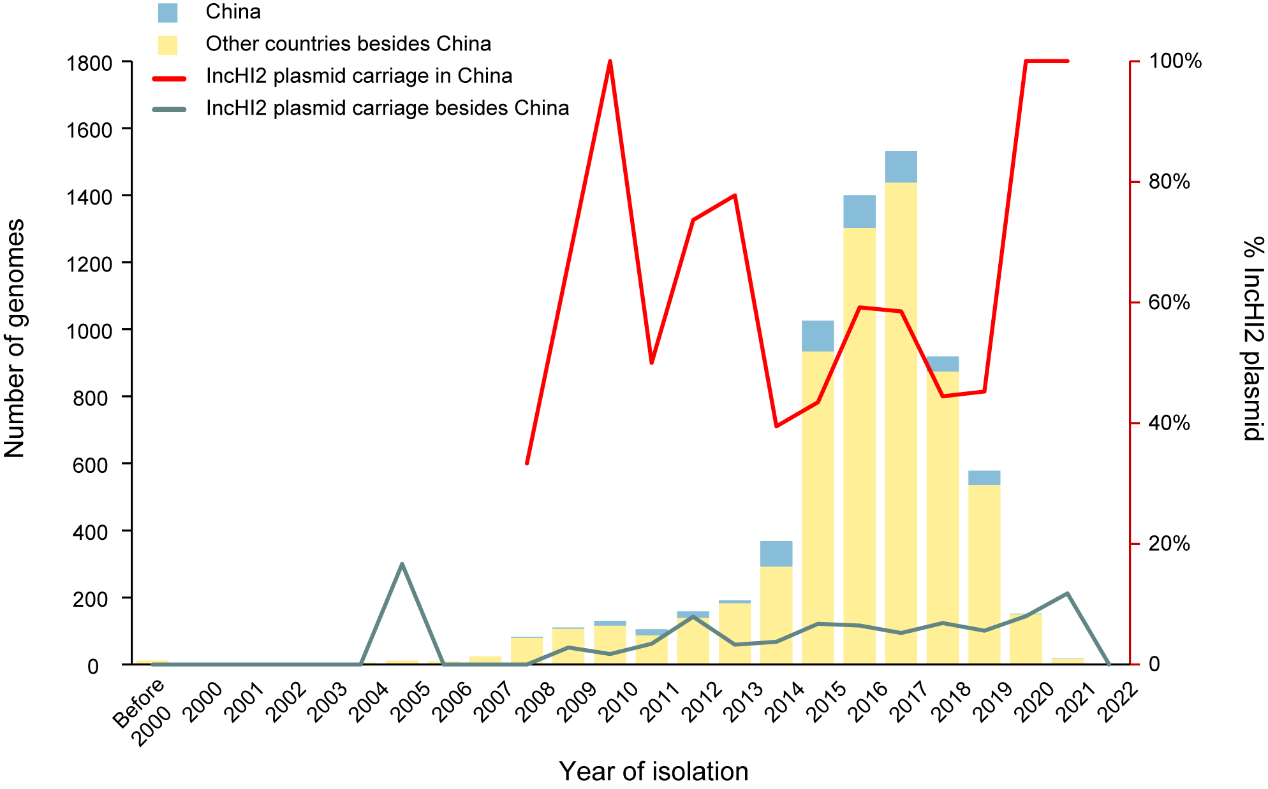
**

**Figure S6.** **The prevalence trend of IncHI2 plasmid carriage in *S*. 4,[5],12:i:- strains**

**
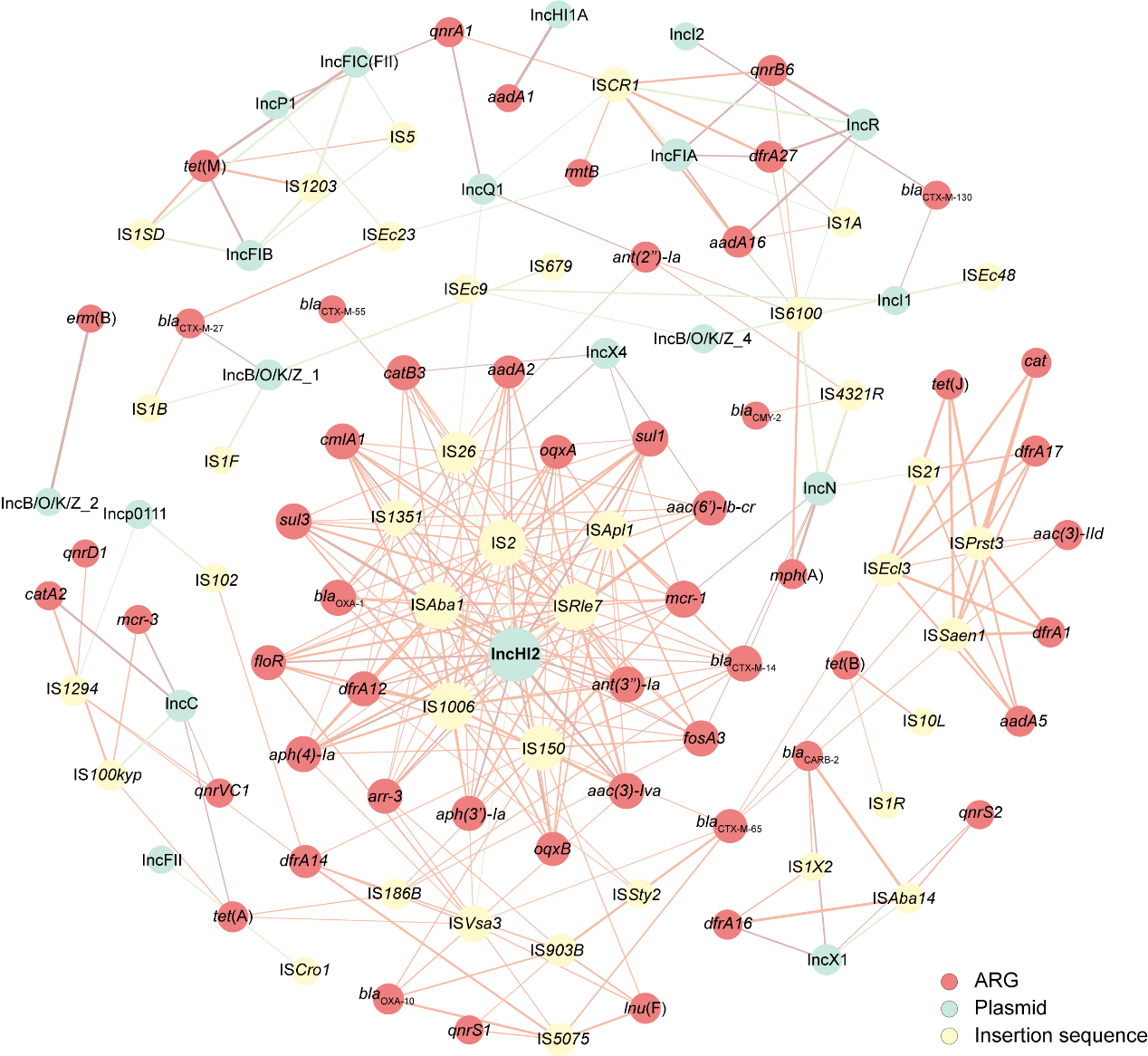
Figure S7.** **The network graph describing the co-occurrence pattern of ARGs with ISs and plasmid replicons within a dataset comprising 344 clinical and 59 animal ST34 *S*. 4,[5],12:i:- strains**

**
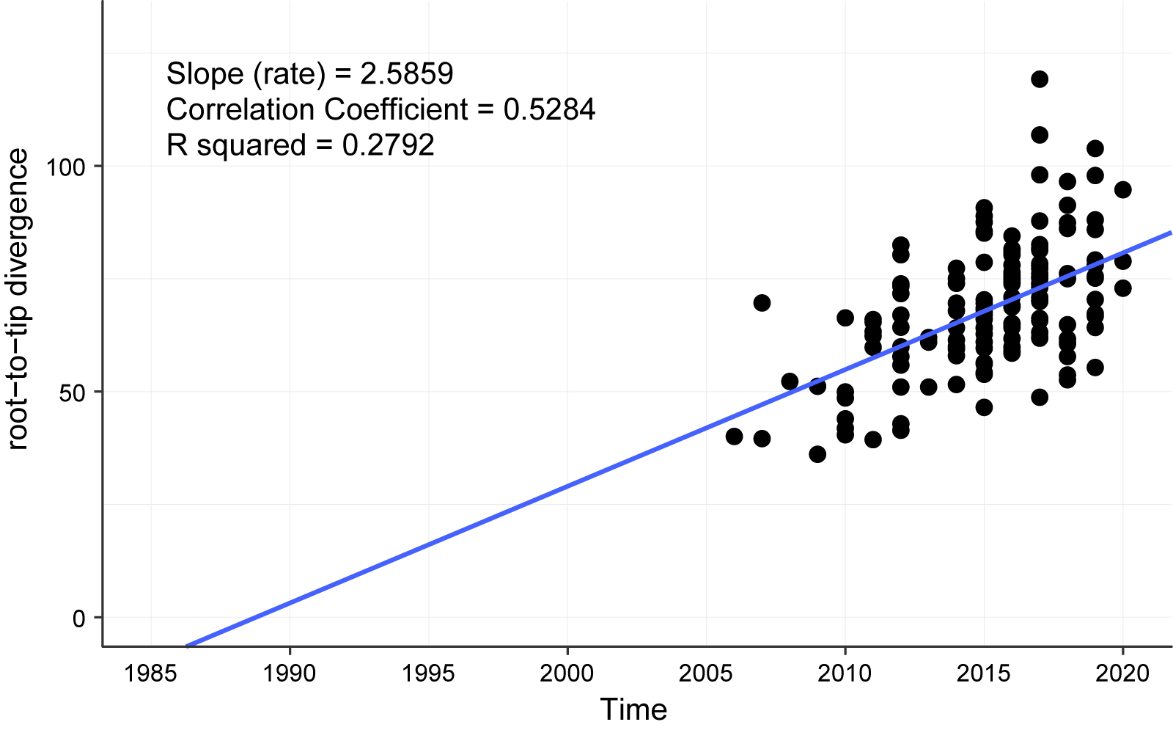
**

**Figure S8.** **Correlation between root-to-tip distance (ML phylogeny) and sampling year for ST34 *S*. 4,[5],12:i:- strains (n = 155)**


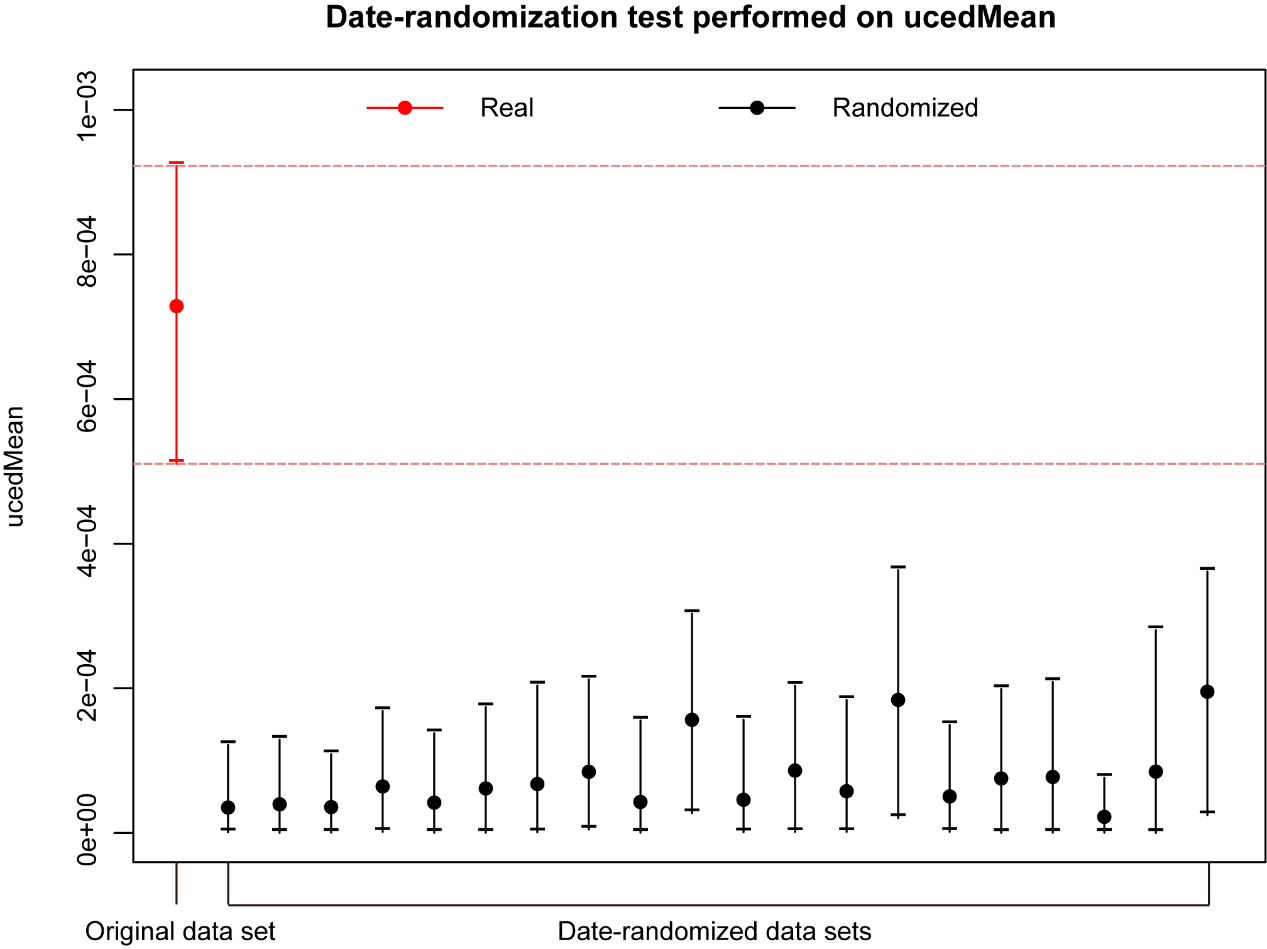


**Figure S9.** **Temporal signal amongst ST34 *S*. 4,[5],12:i:- genomes**

Mutation rate estimates and 95% HPD intervals for BEAST runs with real dates (red) and randomized dates (n=20, black).

**
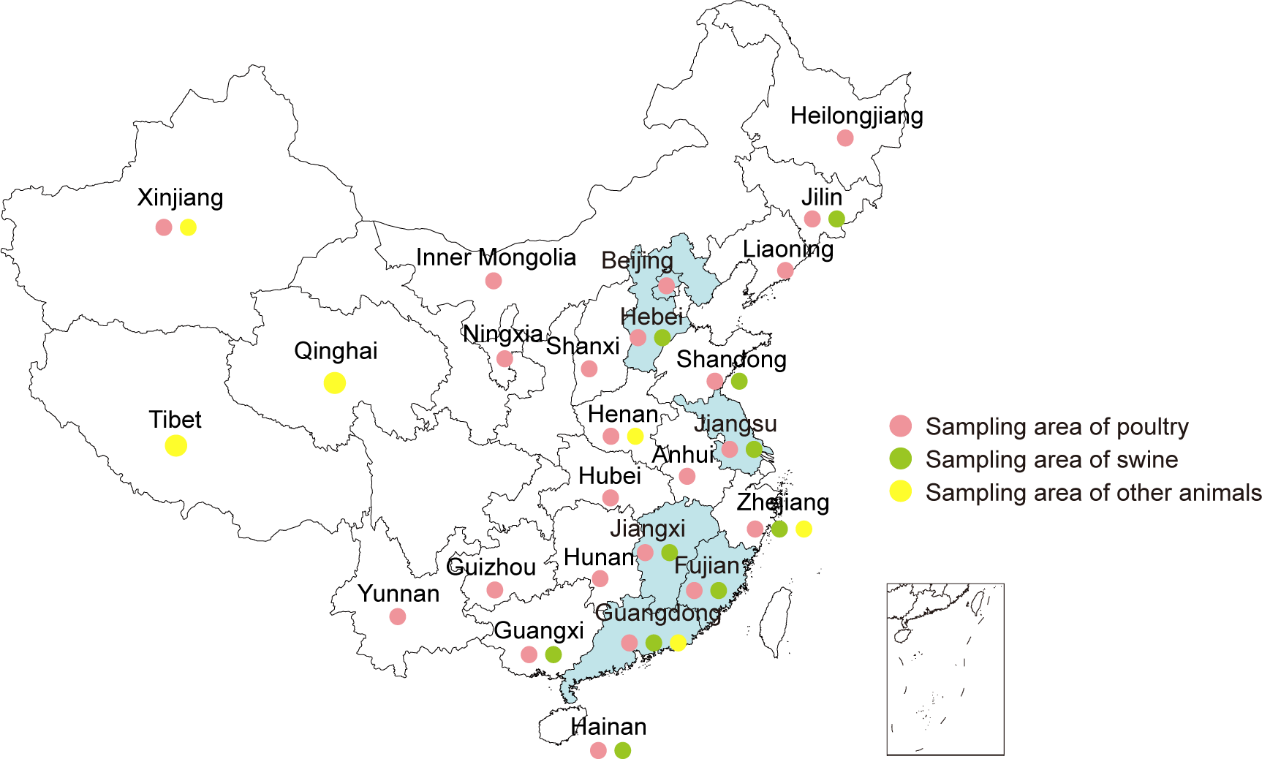
**

**Figure S10.** **Geographic distribution of *Salmonella* 4,[5],12:i:- isolates from animals across 6 provinces in China**

The *Salmonella* sampling areas of swine, poultry, and other animals are denoted with pink, green, and yellow circles, respectively. The distribution of *Salmonella* 4,[5],12:i:- isolates in China is shaded in light blue.

**
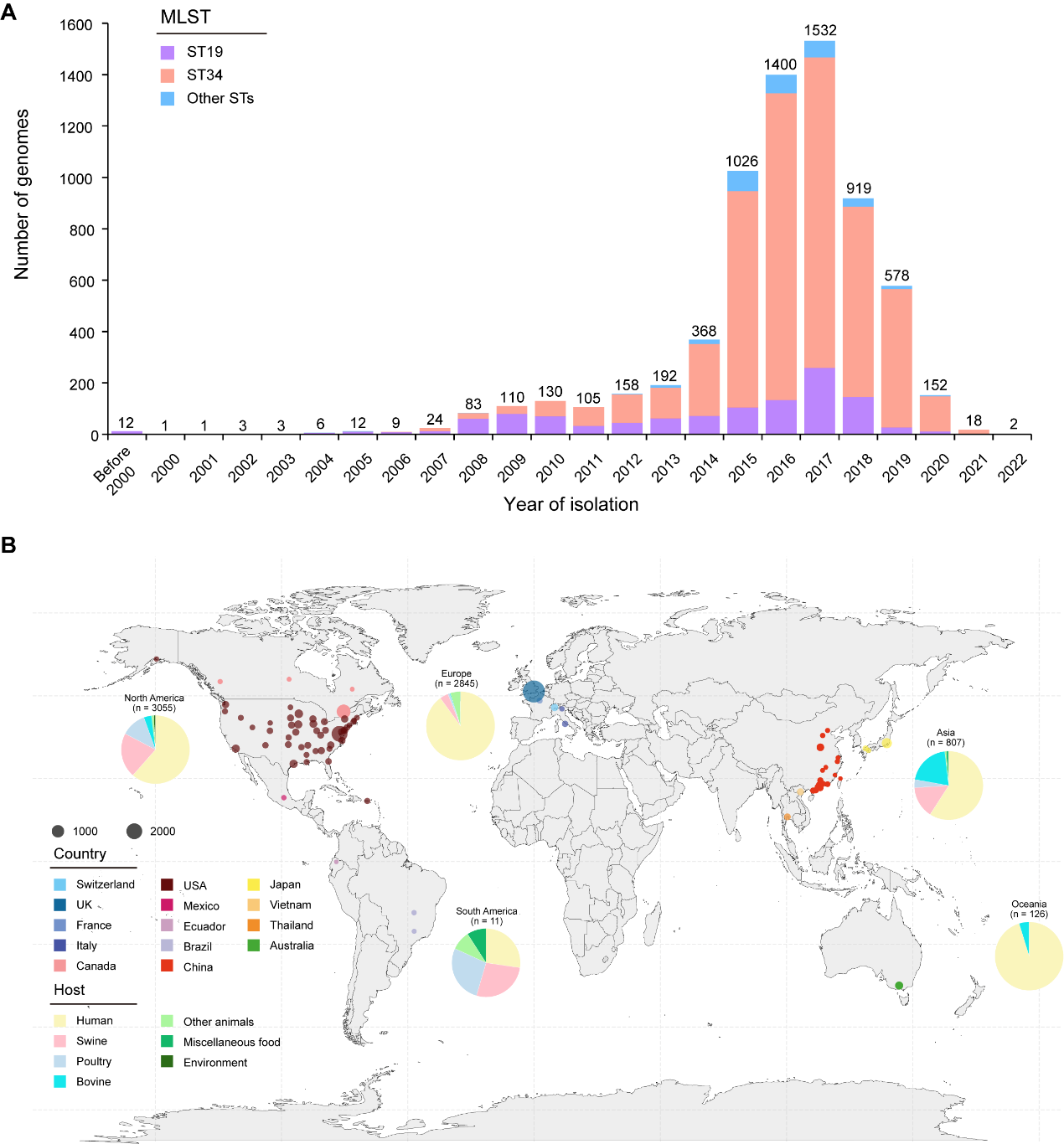
Figure S11.** ***S*. 4,[5],12:i:- genome collection metadata**

Summary of metadata of 352 clinical and 59 animal *S*. 4,[5],12:i:- strains from this study and 6433 strains documented in public database.

**(A)** Year of *S*. 4,[5],12:i:- genome collection coloured according to the multi-locus sequence type (ST);

**(B)** Geographic distribution of *S*. 4,[5],12:i:- assemblies. Points are coloured according to geographic region, the size reflects the number of samples in that region. Distribution of genome isolation source were visualization as pie chart.
